# Limb ischemia in patients with COVID-19

**DOI:** 10.1590/1677-5449.210004

**Published:** 2021-06-09

**Authors:** Julio Cesar Peclat de Oliveira, Walter Jr. Boim Araujo, Sergio Quilici Belczak, Fabiano Luiz Erzinger, Lucas Maia Peclat de Oliveira, Marcos Arêas Marques, Lucas Mansano Sarquis, Bianca Gutfilen

**Affiliations:** 1 Universidade Federal do Rio de Janeiro – UFRJ, Rio de Janeiro, RJ, Brasil.; 2 Universidade Federal do Paraná – UFPR, Curitiba, PR, Brasil.; 3 Instituto de Aprimoramento e Pesquisa em Angiorradiologia e Cirurgia Endovascular – IAPACE, São Paulo, SP, Brasil.; 4 Hospital Erasto Gaertner, Curitiba, PR, Brasil.; 5 Universidade Federal do Estado do Rio de Janeiro – UNIRIO, Rio de Janeiro, RJ, Brasil.; 6 Universidade do Estado do Rio de Janeiro – UERJ, Rio de Janeiro, RJ, Brasil.; 7 Hospital do Trabalhador, Curitiba, PR, Brasil.

**Keywords:** SARS-CoV-2, COVID-19, vascular diseases, endovascular techniques, anticoagulants, embolisms and thrombosis, SARS-CoV-2, COVID-19, doenças vasculares, técnicas endovasculares, anticoagulantes, embolia e trombose

## Abstract

This narrative review covers the life-threatening thromboembolic events associated with SARS-CoV-2 infection/COVID-19. It addresses the physical changes that cause vascular and arterial damage to limbs, laboratory management of coagulation, and management of anticoagulation. COVID-19’s relationship with deep venous thrombosis and arterial thrombosis is also emphasized. The main thromboembolic events described in the literature are illustrated with examples from our experience with COVID-19 patients.

## Resumo

Esta revisão narrativa abrange os eventos tromboembólicos com risco de vida associados a infecção por SARS-CoV-2/COVID-19. Aborda as mudanças físicas que causam danos vasculares e arteriais aos membros, o manejo laboratorial da coagulação e o manejo da anticoagulação. A relação de COVID-19 com trombose venosa profunda e trombose arterial também é enfatizada. Os principais eventos tromboembólicos descritos na literatura são ilustrados a partir de nossa experiência com pacientes COVID-19.

## INTRODUCTION

The relationship between systemic viral infections (H1N1, HIV, and hepatitis) and occurrence of inflammation and hypercoagulation has been previously reported in the medical literature.^1^,^2^ Such infections can alter normal hemostasis due to changes in the coagulation cascade, platelet function, and fibrinolytic system. The resulting hypercoagulability can be caused by increasing procoagulatory factors or direct endothelial injury with increased expression of tissue factor. A viral infection can also induce production and release of procoagulatory microparticles and increase platelet adhesion, increasing the incidence of thromboembolic events.^3^-^6^

SARS-CoV-2 is associated with a broad spectrum of respiratory syndromes that can manifest from mild upper airway symptoms to an alveolar microthrombosis with a high mortality rate.^7^ Clinically, more severe patients have dyspnea and progressive hypoxemia, and generally need mechanical ventilatory support. In computed tomography images, the lung parenchyma manifests ground-glass opacity, especially in the periphery. Histologically, from the initial phase, diffuse alveolar edema, hemorrhage, and intra-alveolar deposition of fibrin are present in the most severe patients who develop the Acute Respiratory Distress Syndrome – ARDS.^8^

In patients with ARDS, formation of microthrombi and fibrin deposits in the microcirculation are observed and may be responsible for the change in gas exchange and, consequently, for the deteriorating ventilation-perfusion ratio. Diffuse presence of thrombotic microangiopathy is seen in histological analyses of the alveoli. Compared to patients with influenza, COVID-19 patients have nine times more microthrombi in alveolar capillaries and develop 2.7 times more new vessels through an intussusceptive angiogenesis mechanism.^9^,^10^ Endothelial cell injuries and diffuse microvascular thrombosis suggesting thrombotic microangiopathy are also reported in other organs and may explain the acute onset of multiple organ failure without obvious etiology.^11^,^12^

## MATERIALS AND METHODS

There was no opportunity to present Free and Informed Consent Forms (CIF) during data collection, since the study was conducted in an emergency scenario, in the middle of a pandemic, and because of isolation and the restricted contact between the medical team and family members. Many patients (the ones illustrated here are just some examples) were not followed up by the team, but by their treating physicians and many were from other towns.

### Physiopathology

**Figure 1:**
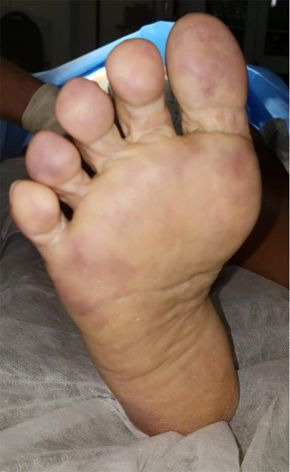
Female patient, 62 years old, hospitalized due to COVID-19, developed an ischemic plaque and possible infectious spot in the left heel, and erythrocyanosis of the forefoot. She was given clinical treatment with full IV heparinization and venous prostaglandin for three weeks. Her clinical condition improved, and she was discharged after 30 days.

Many SARS-CoV-2 infected patients show elevated serum D-Dimer (DD) levels and cutaneous changes in extremities suggestive of thrombotic microangiopathy. Our own experience regarding COVID-19 patients with thrombotic microangiopathy is illustrated in Figure 1. This image is from a 62-year-old female who was admitted to hospital due to COVID-19, developing an ischemic plaque and possible infectious spot in the left heel and erythrocyanosis of the forefoot. She received clinical treatment with full IV heparinization and venous prostaglandin for 3 weeks. Her clinical condition improved and she was discharged after 30 days. Occurrence of disseminated intravascular coagulation (DIC) and large vessel thrombosis have been associated with multiple organ failure in several patients. In an observational analysis of infected patients in Wuhan province (China), significantly higher mortality was found in patients who had increased fibrin degradation products on admission, including D-Dimer (DD), prothrombin time (PT), and activated partial thromboplastin time (aPTT).^10^-^13^

This correlation in critically ill patients between infectious complications and inflammatory activation, with systemic activation of coagulation leading to DIC, is multifactorial. Microorganisms and their components induce expression of several substances, including tissue factor in monocytes and macrophages, which bind to immune cell receptors. Inflammatory activation of the host also results in increased production of proinflammatory cytokines with pleiotropic effects, including activation of coagulation, which can lead to consumption coagulopathy.^8^,^14^

In the early stages of COVID-19, changes in coagulation test results are frequent, although they generally do not result in clinically evident bleeding. It is still unknown which of these changes predict progression to more severe coagulopathies; many factors, including treatment modalities, can influence this unfavorable course.^8^

The acronym CAC (coagulopathy associated with COVID-19) has been used to describe these patients’ coagulation abnormalities. Based on current evidence, the virus does not appear to have an intrinsic procoagulatory effect. The changes seen in coagulation result from several pathways triggered by an intense inflammatory response caused by the virus, the so-called cytokine storm. Polyphosphates derived from microorganisms activate platelets, mast cells, and coagulation factor XII. Circulating serum proteases, including antithrombin, C1 esterase inhibitor, and protein C, tend to decrease during the inflammatory response to infection.^15^

Another critical effect is activation of the complement system, contributing to activation of coagulation factors. Likewise, the inflammatory effect of cytokines can result in vascular activation of endothelial cells, endothelial injury, and prothrombotic activity. This process of endothelial injury can cause thrombocytopenia and reduce levels of natural anticoagulants, while the large-scale hemostatic activation predisposes to occurrence of DIC.^16^-^18^

Therefore, strategies that inhibit these responses can benefit critically ill patients at high risk of death, and the success of these interventions depends on the time at which these therapies are initiated and on the course of the infection.

### Laboratory management of coagulation in patients with COVID-19

Hospitalized patients with recent confirmation or suspicion of SARS-CoV-2 infection should be tested for coagulation assessment at hospital admission, including DD, PT, aPTT, fibrinogen, and platelet count. These tests can provide useful information for therapy and prognostic prediction. Seven to 11 days after the onset of symptoms, increased DD is associated with worse prognosis and a rapid fall in fibrinogen is associated with occurrence of DIC.

There is also a correlation between the period when DD, PT, and aPTT increase and fibrinogen and platelet count decrease and the time of hospitalization. It usually occurs between the seventh and tenth days after admission, although DD can start to rise after four days in some cases. In patients who progress to sepsis, the progression of these changes in laboratory coagulation tests may indicate development of DIC.^19^,^20^

In short:^21^

1. Coagulopathy manifests with increased fibrinogen and DD, and minimal changes in PT, aPTT, and platelet count in the early stages of infection;
2. Increasing interleukin 6 levels are correlated with increased fibrinogen levels;
3. Coagulopathy appears to be related to the severity of the disease and consequent inflammation and not to intrinsic viral activity;
4. Increased DD at hospital admission is associated with increased mortality;
5. The increase in DD after hospital admission precedes multiple organ failure and DIC: (a) observed on the fourth day after admission in non-survivors; (b) longer length of hospital stay is associated with increased DD and development of sepsis physiology.

### Management of anticoagulation in patients with COVID-19

Most of the time, most patients with COVID-19 requiring hospitalization due to respiratory complications present a hypercoagulable state. Compression ultrasound screening for deep vein thrombosis (DVT) is useful. These patients need drug-based prophylaxis for venous thromboembolism (VTE) unless they have absolute contraindications. Considerations regarding pharmacoprophylaxis for these patients are shown in Table 1. Risk assessment of these patients is essential to identify patients at very high risk (Caprini score >8 or Padua score >4), since there may be indications for higher dosage pharmacoprophylaxis.^22^-^27^

**Table 1:** Considerations on prophylactic anticoagulation in COVID-19 patients.

Low-molecular-weight heparin (LMWH): Consider 30 mg twice daily or 40 mg once daily with standard adjustments for renal failure or obesity patients.
Obese patients: (Body Mass Index [BMI] >30) at high risk (Caprini score >8): consider double the usual anticoagulation dose LMWH 60mg from once daily to twice daily. If there is a severe renal failure (ClCr <30 mL/min) or acute renal failure, consider Unfractionated Heparin (UFH) 5,000 IU, subcutaneous, three times a day.
If there is a history of or concern about the occurrence of heparin-induced thrombocytopenia, use fondaparinux.
If the platelet count is less than 30,000 mm^3^, there is significant bleeding, or contraindication to anticoagulation, use mechanical compressive methods.
Direct Oral AntiCoagulants (DOACS) should not be used as prophylaxis in hospitalized patients.

Before patients are discharged, a new assessment must be performed using the Caprini score and patients with scores >8 should benefit from extended pharmacoprophylaxis with unfractionated heparin (UFH), low molecular weight heparin (LMHW), fondaparinux, or direct oral anticoagulants (DOACs), taking care to assess drug interactions, especially if using retrovirals.^25^,^26^

In patients with confirmed venous or arterial thrombosis, full anticoagulation should be instituted following current protocols to treat these pathologies. However, currently, full anticoagulation is also considered for patients without thrombosis, but with significantly increased DD. Some centers consider the criterion of three times the expected value for age, and others separately consider the value >3,000 ng/ml (Table 2). Some studies have shown that patients treated with anticoagulation had reductions in DD levels, more remarkable clinical improvement, and lower mortality than patients who were not anticoagulated.^28^

**Table 2:** An approach based on bedside ultrasound screening dividing patients into three categories.

Category	D-Dimer	Treatment
I	< 3.000 ng/mL and no evidence of venous thromboembolism (VTE)	Patients receive standard deep vein thrombosis (DVT) prophylaxis (enoxaparin 40 mg once a day) and are monitored with serial D-Dimer (DD) testing
II	> 3.000 ng/mL and negative ultrasound	Patients receive intensified prophylaxis against deep vein thrombosis (DVT) (enoxaparin 40 mg every 12 hours)
III	Confirmed thrombosis	Full anticoagulation

### COVID-19 and DVT

DVT patients with COVID-19 usually have dyspnea, hypoxemia, and homodynamic instability; VTE can be neglected in this clinical condition. The exact pathophysiology of extensive vessel thrombosis in COVID-19 is still under investigation and can be attributed to several factors:^20^,^29^

- Cytokine storm induced by SARS-CoV-2 infection activating the coagulation cascade: pro-inflammatory cytokines such as interleukin (IL) 1β and IL-6 stimulate expression of tissue factor in immune cells and initiate activation of the extrinsic coagulation pathway;
- Suppression of the fibrinolytic system due to decreased activity of urokinase-type plasminogen activator and increased release of plasminogen activator inhibitor-1;
- Platelet activation by various pro-inflammatory cytokines and rapid attachment of platelets to the damaged endothelium;
- Endothelial damage induced by inflammation further accelerates the thrombotic reaction;
- Venous stasis due to immobilization in critically ill patients.

These factors can be aggravated by hypoxia, which decreases the anticoagulant properties of endothelial cells and increases the permeability and adhesion of leukocytes by secondary ischemia-reperfusion injury.

Hypoxia can trigger oxidative stress through reoxidation of endothelial cells, promote production of superoxide, and inhibit nitric oxide production, resulting in damage to endothelial cells, with an imbalance of fibrinolysis, contributing to a pro-coagulant state.^7^

There was a high incidence of DVT and PE in hospitalized patients, especially in the Intensive Care Unit (ICU), justifying thromboprophylaxis, as explained above.^20^,^29^,^30^

**Figure 2:**
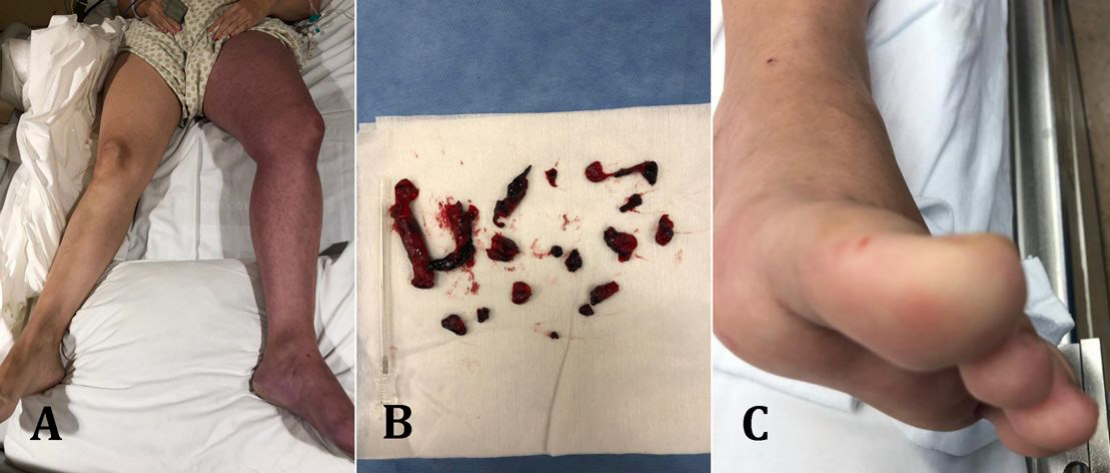
A young female patient using hormonal contraceptives was admitted to the Emergency with COVID-19 and pain and critical edema in the left lower limb, and absence of distal pulses with no other symptoms **(A)**. Emergency Fogarty thrombectomy was successfully performed on the venous iliac femoral segment **(B)**. Postoperatively, she coursed with worsening laboratory tests and pneumonia. She was discharged after 3 weeks and the limb remains healthy, on oral anticoagulation with rivaroxaban **(C)**.

This is illustrated in Figure 2, showing images from a young female COVID-19 patient who was taking hormonal contraceptives and was admitted to the Emergency with COVID-19 and pain and critical edema in the left lower limb and absence of distal pulses with no other symptoms. Emergency Fogarty thrombectomy was successfully performed on the venous iliac femoral segment. She was discharged after three weeks and the limb remains healthy with oral anticoagulation using rivaroxaban.

Initial evidence regarding patients with COVID-19 and diagnosis of DVT in China pointed to worse prognosis, higher rate of ICU admission, and higher mortality compared to patients with COVID-19 but without DVT. There was no difference in mortality regarding the location of the DVT: patients with proximal DVT had the same rate as those with distal DVT.^31^

### COVID-19 and arterial thrombosis

**Figure 3:**
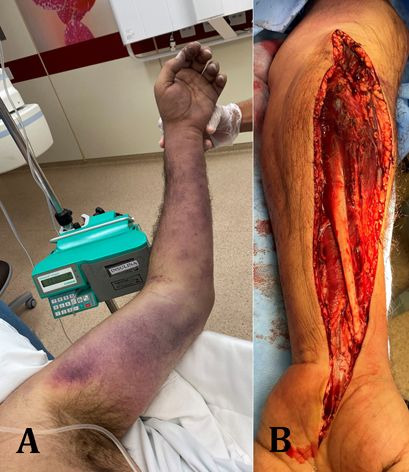
Male patient, 61 years old, with controlled hypertension, hospitalized due to edema and pain in the left upper limb **(A)**, tested positive for COVID-19. Color Doppler ultrasound revealed extensive arterial thrombosis of the left upper limb, absence of flow in radial and ulnar arteries, and palmar arch. The examination showed evidence of forearm compartment syndrome. The patient underwent decompressive fasciotomy **(B)** and arterial Fogarty catheter thrombectomy. He progressed well and was discharged on oral anticoagulants.

Viral inclusions within endothelial cells and mononuclear and polymorphonuclear cell infiltration have been demonstrated, evidencing endothelial apoptosis in a COVID-19 post-mortem study. Microcirculatory dysfunction, in addition to systemic hypercoagulability and microvascular endothelial injury, generates thrombotic microangiopathy. This “endotheliopathy” may explain reports of cerebrovascular complications in younger patients, myocardial ischemia, and increasing reports of microcirculatory and macrocirculatory thromboembolic complications.^21^-^33^ This presentation is illustrated in Figure 3, showing images from a 61-year-old, male COVID-19 patient, with extensive thrombosis of the left upper limb, and absence of flow in radial and ulnar arteries and the palmar arch. He underwent decompressive fasciotomy and Fogarty catheter thrombectomy, recovered well and was discharged on oral anticoagulants.

This coagulopathy associated with COVID-19 can manifest as acute myocardial infarction, ischemic stroke, and/or acute limb ischemia, not only in elderly patients, but also in younger patients without comorbities,^34^ during the second and third weeks of illness, increasing mortality rates.^35^-^37^

It is not clear whether antiphospholipid antibodies in SARS-CoV-2 infection are part of an epiphenomenon or are involved in the genesis of thrombolytic events, which should be suspected in the presence of acute and extensive arterial thrombosis in young, otherwise healthy patients with COVID-19. In the presence of known preexisting antiphospholipid antibodies, additional care should be taken, observing these patients for venous and arterial thromboembolic phenomena.^38^

## CONCLUSIONS

Health professionals should be aware of the life-threatening thromboembolic events associated with COVID-19, so that immediate and appropriate interventions can be performed.
